# Cytogenetic and molecular characterization of an atypical ETP-ALL case with BCL2 dependency: therapeutic implications for Venetoclax use

**DOI:** 10.1007/s11033-025-10979-1

**Published:** 2025-09-18

**Authors:** Francesco Gigliotti, Carolina Brescia, Salvatore Audia, Maria Eugenia Gallo Cantafio, Paola Malatesta, Michelle‑Li Bellisario, Roberta Torcasio, Renato Cantaffa, Eulalia Galea, Rodolfo Iuliano, Giuseppe Viglietto, Francesco Trapasso, Maria Concetta Galati, Nicola Amodio, Rosario Amato

**Affiliations:** 1 Renato Dulbecco University Hospital, Catanzaro, Italy; 2 Dept. of Health Science, Medical School, University “Magna Graecia”, Catanzaro, Italy; 3 Department of Experimental and Clinical Medicine, Medical School, University “Magna Graecia”, Catanzaro, Italy; 4 Medical Genetics Unit, Dept. of Health Science, Medical School, University Hospital “Mater Domini”, University “Magna Graecia”, Catanzaro, Italy

**Keywords:** ETP-ALL, Pediatric leukemia, Venetoclax, BCL2, Th17

## Abstract

**Background:**

Early T-cell precursor acute lymphoblastic leukemia (ETP-ALL) is a rare, high-risk subtype of T-ALL characterized by distinctive immunophenotypic and genomic features. It is often associated with induction failure and frequent relapses. Despite recent advances in its molecular characterization, the prognosis remains dismal, and effective targeted therapies are limited.

**Methods and results:**

We report a pediatric, multi-refractory ETP-ALL case with novel cytogenetic alterations, including a 4q deletion and a t(16;18)(q24;q21) translocation. Molecular profiling revealed progressive activation of the BCL2 pathway and disruption of Th17-related immune markers. Ex vivo sensitivity assays performed at different disease stages demonstrated increasing BCL2 dependency. Based on these findings, venetoclax was administered on a compassionate-use basis, resulting in rapid hematologic recovery and a marked reduction in blast percentage.

**Conclusions:**

This case highlights the role of clonal evolution and immune deregulation in accompanying BCL2 addiction in relapsed ETP-ALL. Altogether, our findings underscore the therapeutic potential of venetoclax in refractory pediatric ETP-ALL cases with progressive BCL2 dependency.

**Supplementary Information:**

The online version contains supplementary material available at 10.1007/s11033-025-10979-1.

## Introduction

Early T-cell precursor acute lymphoblastic leukemia (ETP-ALL) is a subtype of T-cell acute lymphoblastic leukemia (T-ALL) that was recognized as a distinct clinical and biological entity by the World Health Organization in 2016. Originating from thymic progenitor cells at an early stage of T-cell differentiation, ETP-ALL accounts for approximately 16% of childhood and 22% of adult T-ALL cases [1, 2]. This subtype is characterized by a unique immunophenotypic and genotypic profile that straddles both myeloid and lymphoid lineages. Diagnosis of ETP-ALL is primarily based on immunophenotypic markers, including strong cytoplasmic CD3 and CD7 positivity; absence of CD1a and CD8; reduced or absent CD5 expression; and positivity for myeloid-associated markers such as CD34, CD117, CD65, CD33, CD11b, and HLA-DR [1, 3]. Additional markers, such as cytoplasmic CD2 and, occasionally, CD4, have been identified as helpful in distinguishing ETP-ALL from other leukemic subtypes [3]. ETP-ALL is also known for its complex karyotype and significant genomic instability. Numerous genetic abnormalities have been identified, particularly in the RAS signaling pathway including mutations in *KRAS, NRAS, BRAF, FLT3*, and *IL7R* as well as in transcription factors essential for hematopoietic development, such as *GATA3, RUNX1, ETV6*, and *LMO2*. Furthermore, genes involved in epigenetic regulation, including *DNMT3A, EZH2*, and *EP300*, are frequently altered [2, 4]. Several gene rearrangements have also been reported. These include *MEF2C* rearrangements that drive overexpression of *LMO2* and *LYL1*, and *KMT2A* rearrangements that lead to activation of HOXA-cluster genes, both of which contribute to leukemogenesis [5, 6]. Recent studies have begun to elucidate the critical role of anti-apoptotic signaling pathways in ETP-ALL, with particular attention to the antiapoptotic BCL2 protein. Aberrant expression of BCL2 is frequently observed in high-risk leukemias, including pediatric subtypes [7, 8], in which it contributes to chemoresistance and poor prognosis. In ETP-ALL, elevated BCL2 levels may reflect an intrinsic survival advantage of leukemic stem-like cells and could serve as a dynamic biomarker of disease progression. Furthermore, BCL2 overexpression may not be present at the time of diagnosis but can emerge during clonal evolution or relapse, emphasizing the need for longitudinal molecular profiling. It is therefore imperative to dissect the expression patterns of BCL2 in ETP-ALL, as this is not only crucial for prognostic stratification but also for identifying patients who may benefit from targeted therapies, such as venetoclax, which directly inhibit BCL2-dependent survival mechanisms [9]. Despite the high response rates to conventional chemotherapy, particularly in pediatric patients, a subset of cases exhibits resistance, prompting the investigation of alternative therapeutic strategies [10]. Promising targeted approaches include ruxolitinib, a JAK/STAT pathway inhibitor [11]; hypomethylating agents such as decitabine and azacytidine [10, 12]; and FLT3 inhibitors, which have shown efficacy in certain molecular subtypes [13]. Recently, overexpression of the anti-apoptotic protein BCL2 has been observed in select ETP-ALL cases, suggesting that BCL2 inhibition using venetoclax may represent a viable therapeutic option. However, the biological role of BCL2 in ETP-ALL remains poorly understood, and clinical application of venetoclax remains sporadic and lacks strong molecular basis and integration into standardized treatment guidelines [14]. In this study, we report for the first time novel genetic alterations arising during disease progression that drive BCL2-dependent blast proliferation, which was not evident at diagnosis. An ex vivo assay using patient-derived primary blasts confirmed sensitivity to venetoclax, supporting both the cytogenetic findings and the patient’s subclinical response. Together, the cytogenetic, molecular, and clinical data demonstrate the emergence of BCL2 dependency during disease evolution, providing a rationale for incorporating BCL2-targeted therapies in relapsed or refractory ETP-ALL.

## Materials and methods

### Sample collection

Bone marrow and peripheral blood samples from the patient exhibiting an atypical presentation of ETP-ALL, as well as from a control subject diagnosed with standard ALL-T EGIL-III and non-tumoral control sample were collected at the Oncohaematology Department of the Pugliese-Ciaccio Hospital in Catanzaro. The collection of these samples was conducted in accordance with the standard diagnostic procedures. Informed consent was obtained from all patients (parent or legal guardian for minor subjects) and the study protocol was approved by the Calabria Regional Ethics Committee approval number: V1 n.25 18/10/2023.

### Cytomorphology—May-Grünwald-Giemsa staining

For cytomorphological analysis, slides for each collected sample were produced and stained with May-Grünwald-Giemsa using the Sysmex SP-10 automatic instrument from Dasit. The instrument characteristics were set according to the haematocrit (HCT) of each individual patient.

### Conventional karyotyping

Chromosome analysis was performed on at least 10 metaphases from bone marrow lymphocytes by conventional G-banding techniques (300 band resolution). Karyotypes were obtained from unstimulated lymphocytes, collected after 24 or 48 h, according to standard protocols. The results were described in accordance with the ISCN (International System for Human Cytogenomic Nomenclature) 2020 [15]. Images were captured by DMRA microscope (Leica Microsystems, Germany) with a magnification of 100X and analyzed with CytoVision version 7.3.1 (Leica Biosystems Richmond, Inc.).

### Sample processing

Bone marrow mononuclear cells (BMNCs) were isolated by density gradient centrifugation in Histopaque-1077 separation solution. BMNCs were plated in RPMI-1640 supplemented with a 5% serum solution and a 1% penicillin–streptomycin solution for 12 h in a 37 °C incubator in a humidified atmosphere of 5% CO_2_ and 95% air. After adhesion of monocytes and dendritic cells, BMLs (lymphocyte-enriched bone marrow fraction) were derived, and subsequently frozen or used for experiments.

### RNA extraction and quantitative real-time PCR

RNA extraction from BMLs was performed using NucleoSpin RNA (MACHEREY–NAGEL) according to the manufacturer’s instructions. RNA was then quantified using NanoDrop 2000/2000c spectrophotometer (ThermoFisher) and 500 ng was reverse transcribed using the High-Capacity RNA-to- cDNA kit (Applied Biosystems, CA) according to the manufacturer’s instructions. One microliter of cDNA was amplified via real-time PCR using GoTaq qPCR Master Mix (Promega, USA) and 10 pmol of primers specific for SGK1, RANBP1, IL23R, BCL2, RORγt, STING, FOXP3, CD45, IL17A and HPRT1, the latter used as a housekeeping gene (the primer list can be found in the suppl. table 2). Quantitative real-time PCR assays were performed in triplicate in a total volume of 20 μL using a CFX96 Touch Real-Time Detection System under the following conditions: initial denaturation for 2 min at 95 °C, followed by 40 cycles of 15 s at 95 °C and 1 min at 57 °C. The specificity of the PCR products was determined via melting curve analysis. The fold changes were calculated using the 2^−ΔΔCt^ threshold cycle method. The single-tube TaqMan assays (Applied Biosystems, CA) were used for quantitative analysis of MARCH5 (Hs01546967) and MFN2 (Hs00208382) expression levels. Normalization was performed using human GAPDH (Hs03929097_g1). Quantitative real time PCR was performed in triplicate, including no-template controls, by using QuantStudio 12 K Flex reader (ThermoFisher Scientific). Relative expression was calculated using the comparative cross threshold (Ct) method.

### Immunoblot analysis

BMLs were collected for protein extraction using lysis buffer (50 mM Tris–HCl, pH 7.4, 0.15 M NaCl, 0.5% IGEPAL CA-630, 25 mM NaF, 1 mM DTT, 1 mM Na_3_VO_4_, 2 mM PMSF and 8 nM Aprotinin). The cell lysates were separated via SDS page using type 4–15% Mini-PROTEAN TGX STAIN-FREE precast gels (Bio-rad) and transferred onto a 0.2 mm PVDF membrane via Trans-Blot Turbo Transfer Pack (Bio-Rad). Membrane blocking was performed with 1X TPBS, 0.1% Tween-20 with 5% w/v skimmed milk powder for 1 h at room temperature and incubated overnight with antibodies, namely BCL2(1:1000 Santa Cruz Biotechnology), RANBP1 (1:1000 Santa Cruz), IL23R (1:1000 ThermoFisher), SGK1 (1:1000 Millipore), STING (1:1000 Cell Signaling Technology), C-MYC (1:1000 Santa Cruz), MARCH5 (1:1000 ThermoFisher), Mitofusin-2 (1:1000 Cell Signaling Technology), BIM (1:1000 Cell Signaling Technology), and β-actin (1:10,000 SIGMA), the latter used as loading control (the code of antibodies used is listed in the Suppl. Table 2). All primary antibodies were detected using appropriate HRP-conjugated secondary antibodies. Detection was performed using ECL reagent (Cytiva) and chemiluminescence signals were recorded using Uvitec Cambridge.

### Cell viability assay

Cell viability was assessed by Cell Titer Glo assay (CTG; Promega, USA), according to manufacturer’s instructions. Briefly, T-ALL cell lines and patients-derived ETP-ALL primary cells were seeded in 96 well plate and treated with different concentrations of venetoclax* (*100 nM – 1 µM – 10 µM) for 72 h. Then, cell viability was evaluated by CTG luminometric assay. Luminescence was measured using GloMax multi detection system (Promega, USA). All the experiments were performed in triplicate.

### Statistical analysis

The experiments were expressed as mean ± standard deviation (SD). Differences between two groups were analyzed using unpaired two-tailed Student's t-test. Comparisons between multiple groups were performed using one-way ANOVA, followed by Bonferroni's post hoc test. The analysis was conducted using GraphPad Prism software (San Diego, CA, USA Version 9) and differences were considered significant at *p ≤ 0.05, **p ≤ 0.01 and ***p ≤ 0.001.

## Results and discussion

**Fig. 1:**
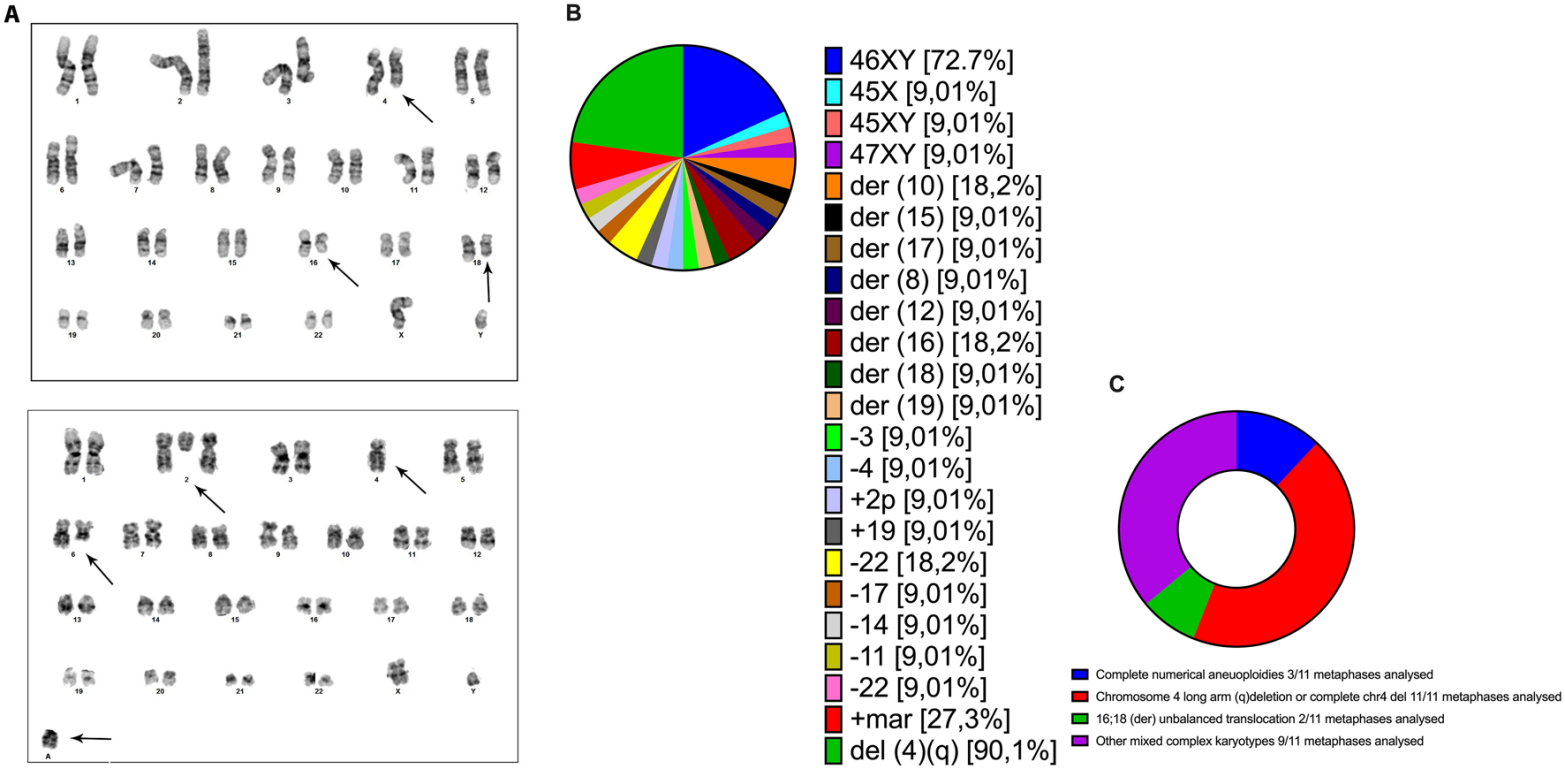
Bone marrow karyotypes at the relapse stage.** A:** Bone marrow karyotypes showing the main pathological features observed using conventional G-banding techniques (300-band resolution). Unstimulated lymphocytes were harvested at 24 and 48 h to obtain karyotypes following standard protocols. Results were reported according to the ISCN 2020 guidelines. **B**: Representative karyotype formulas based on the observed metaphases. **C**: Relative frequency of the key detected abnormalities. The percentages shown in the pie charts were calculated based on the different chromosomal abnormalities, divided by the number of metaphases observed (11) **(B-C)** and then converted into percentages** (B)**

**Fig. 2:**
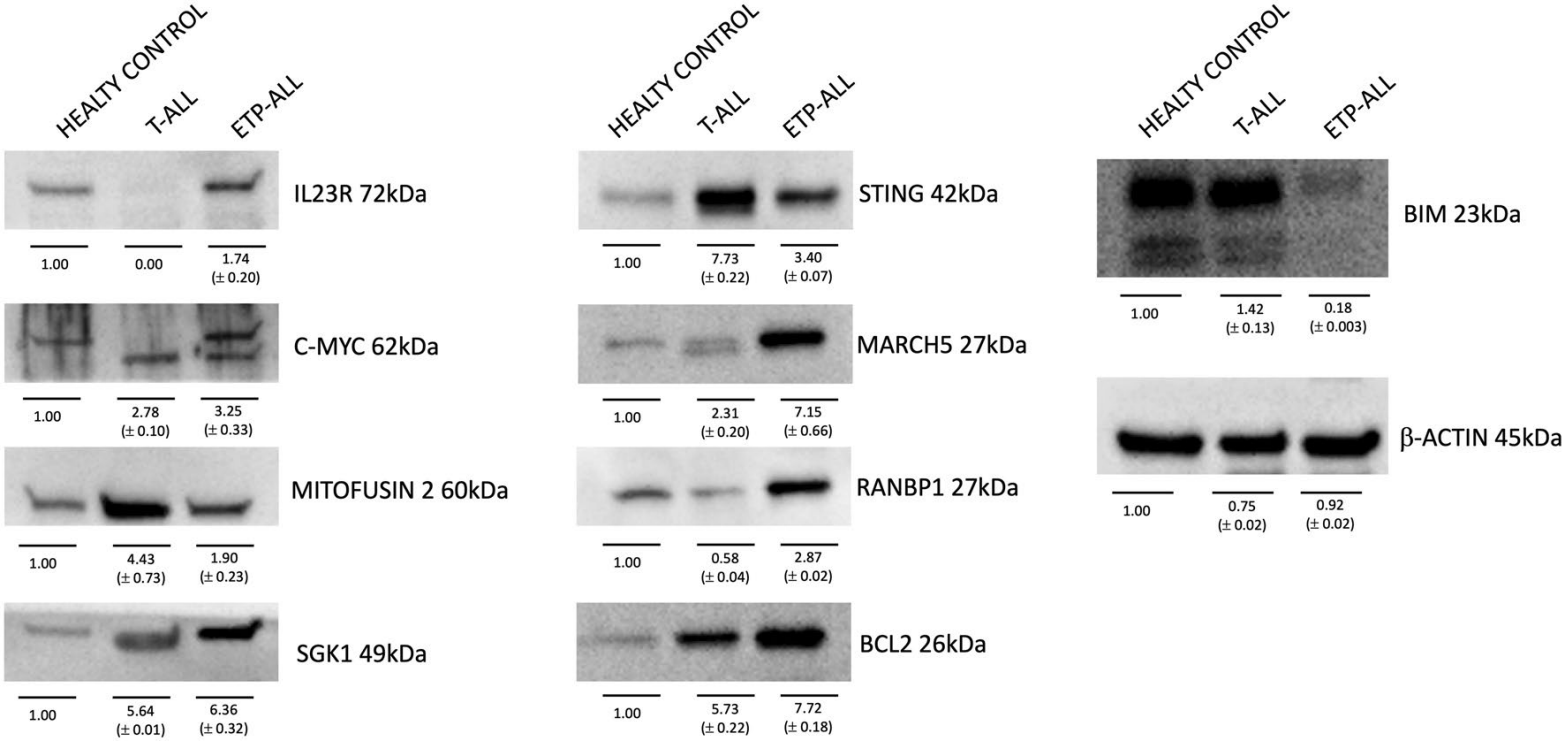
Molecular analysis of markers of BCL-2 dependency**.** Immunoblot analysis of C-MYC, MITOFUSIN-2, MARCH5, BCL2, BIM-associated BCL2-dependent signaling markers and IL23R, SGK1, RANBP1 and STING-associated Th17^+^ markers in primary bone morrow lymphocyte, derived from a healthy control, classical T-ALL and ETP-ALL. Equal loading was verified by means of β-ACTIN. (n = 3). The densitometric expression analysis was processed using ImageJ software

**Fig. 3:**
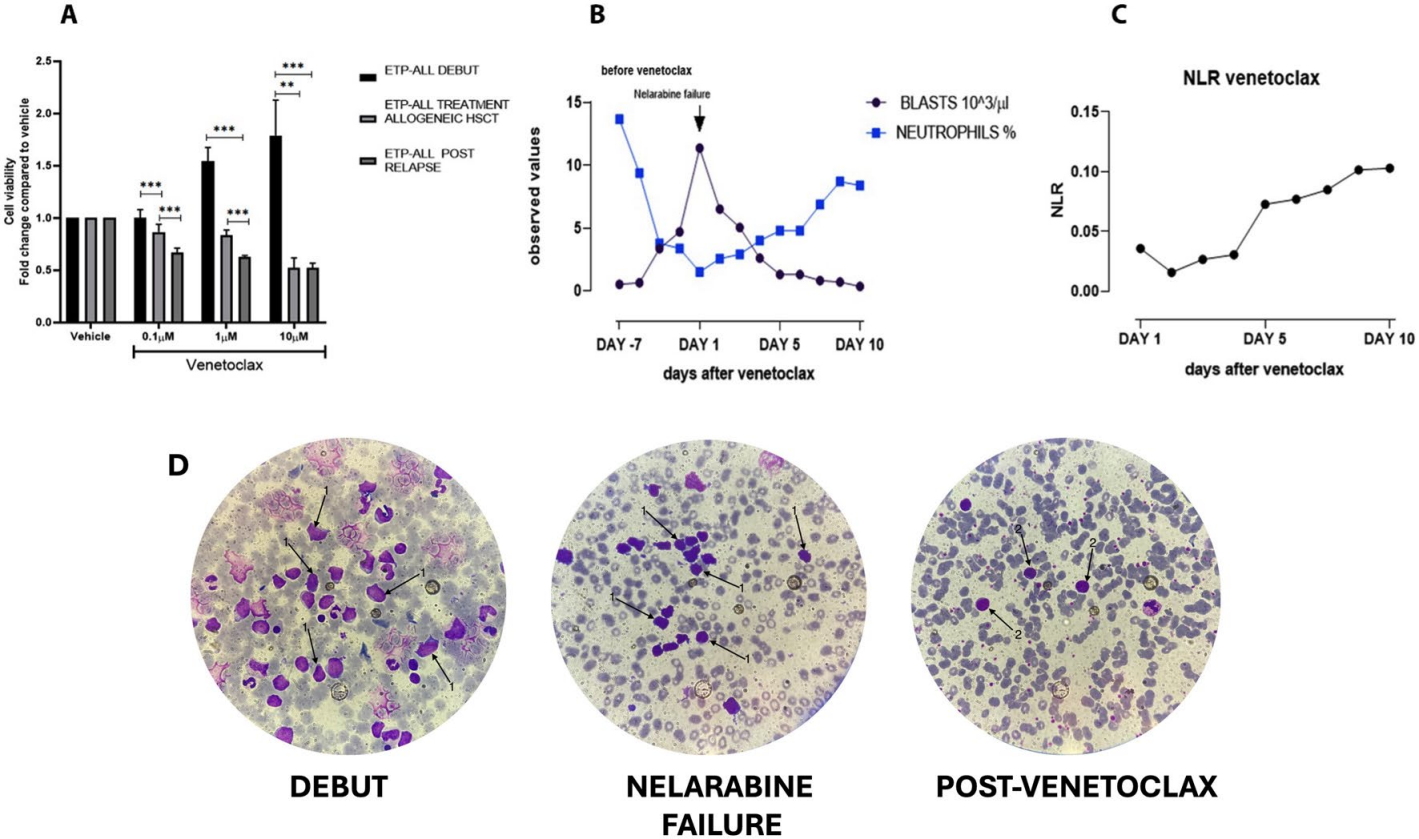
Preclinical and clinical evaluation of venetoclax therapeutic activity**. A:** venetoclax sensitivity was assessed in ETP-ALL disease stages (debut, post allogeneic HSCT, and relapse) using escalating doses of venetoclax (0.1,1 and 10 µM), by CTG assay. Data are representative of at least three independent experiments and are shown as mean ± SD. *p ≤ 0.05, **p ≤ 0.01 and ***p ≤ 0.001 determined by one-way ANOVA followed by Bonferroni’s post hoc test. **B:** trend curves of blasts (10 ^3^/µl) and neutrophils (%) before and after introduction of salvage therapy with venetoclax 100 mg/die/os. **C:** NLR ratio curve derived from the first ten days of venetoclax therapy (100 mg/die/os). **D:** bone marrow smears exemplifying bone marrow cytology in the debut phase, nelarabine failure, and after venetoclax treatment, stained with May-Grünwald-Giemsa using the Sysmex SP- 10 automatic instrument from Dasit. Label 1 indicates leukemic blasts; label 2 indicates physiological lymphocytes

A 14-year-old male presented with a two-week history of persistent vomiting and abdominal pain. Imaging revealed hepatosplenomegaly, lymphadenopathy, and testicular lesions. Bone marrow aspiration showed 51% blasts composed of medium-sized cells with a high nuclear-to-cytoplasmic ratio and a distinctive immunophenotype: cyCD3 + + +, CD7 +, CD2 +, CD8 +, CD33 +, CD34 +, CD117 + +, CD38 + + +, CD56 + +, HLA-DR +, and negative for CD1a, CD5, and CD13 (Table 1) [3]. The diagnosis of ETP-ALL (EGIL T-I/II) was established [10, 16], and the patient was enrolled in the AIEOP-BFM ALL 2017 protocol [17]. Despite an initial reduction in peripheral blasts, MRD remained elevated at day 15 (20%) and day 33 (18%), leading to high-risk stratification [17]. Following intensified chemotherapy according to AIEOP-BFM ALL 2017 (Suppl. Table 1) and allogeneic HSCT from a matched sibling donor, relapse occurred within six weeks. At relapse, 91% of marrow blasts exhibited an altered immunophenotype (Table 1; Suppl. Figure 2B). Compassionate use venetoclax therapy was then initiated, resulting in significant clinical improvement within 10 days. For the purpose of establishing a control group, clinical history, bone marrow tissue and biological and biochemical markers were examined for a subject of the same age diagnosed with EGILIII T-cell leukemia. The basis for the classification of T-ALLs by the European Group on Immunological Classification of Leukemia (EGIL) was formed by knowledge of the expression of immunological markers during differing stages of T-cell growth. In accordance with the EGIL classification, T-ALLs were delineated as CD3-positive and TdT-positive. A further subclassification was then made, into the following categories, on the basis of differing immunophenotypic profiles: T-I (pro-T-ALL); T-II (pre-T-ALL); T-III (cortical-T-ALL); and T-IV (mature T-ALL). Despite the fact that EGIL classification did not reveal a clearly defined category of ETP-ALL, it was hypothesized that cases of T-I ALL and a proportion of T-II ALL could in fact represent ETP-ALL, thus motivating the decision to undertake a comparison between ETP-ALL and a case of T-ALL EGIL III [10, 18]. At diagnosis, karyotype was normal, with no detectable common rearrangements (e.g., BCR/ABL1, ETV6/RUNX1). At relapse, extensive chromosomal abnormalities emerged, including 27.2% metaphases with numerical aneuploidy and deletions on chromosome 4q (Fig. 1C). Notably, an unbalanced t(16;18) translocation affecting BCL2 was identified in 18.2% (2/11) of metaphases (Fig. 1A, B, C), a rare but pathogenic event in ETP-ALL, potentially sustaining BCL2 overexpression and clonal persistence [19]. In light of the severe bone marrows showing hypocellularity at the time of recurrence, only 11 metaphases could be thoroughly analysed. While this does limit formal confirmation of clonality according to the International System for Cytogenetic Nomenclature (ISCN) criteria, the detection of recurrent aberrations was nevertheless facilitated. This included the t(16;18) translocation involving *BCL2*, whose pathogenic relevance in T-ALL provides compelling evidence of its biological non-randomness, notwithstanding the limited number of metaphases analyzed [20]. Notably, expression analysis revealed markedly elevated BCL2 protein levels in relapsed ETP-ALL compared to classical T-ALL and healthy controls (Fig. 2). Conversely, MFN2 a BCL2-negative regulator [21–23] was downregulated, while MARCH5, which inhibits MFN2, was overexpressed (Fig. 2) [24]. This axis, alongside increased c-MYC and loss of the pro-apoptotic BIM, supports a BCL2-dependent, apoptosis-resistant phenotype [25, 26]. Additional investigation of Th17-associated differentiation markers [27, 28], linked to leukemic resistance, revealed upregulation of IL23R, SGK1, and RANBP1, and decreased STING, an immune-activating factor [29–32] (Fig. 2). Notably, although protein levels increased, mRNA transcripts were found downregulated (Suppl. Figure 1A, B, C). The discordance between the levels of mRNA and protein of Th17-associated immune markers in ETP-ALL blasts may be indicative of post-transcriptional regulatory mechanisms. Such mechanisms may include altered mRNA stability, translational efficiency, or proteasomal degradation. These mechanisms may be selectively co-opted during leukemic clonal evolution to sustain differentiation blockade and immune escape [33–35]. Given cytogenetic and molecular evidence of BCL2 pathway activation, ex vivo venetoclax sensitivity assays were performed across three disease stages: diagnosis, post-HSCT, and relapse. While resistant at diagnosis, blasts displayed progressive venetoclax sensitivity post-transplant, peaking at relapse (Fig. 3A). In view of the evidence presented and the comprehensive clinical assessment, compassionate use venetoclax (100 mg/day orally) was approved. Within 10 days, clinical parameters, such as blast reduction, neutrophil recovery, and normalization of hematologic indices (Fig. 3B, C), markedly improved. Bone marrow morphology confirmed remission induction, shifting from extensive blastic infiltration (Fig. 3D, MIDDLE and Suppl. Figure 2) to reconstitution with mature lymphocytes and normalized cytomorphology (Fig. 3D, RIGHT).

**Table 1:** ETP-ALL characterized by positivity of cyCD3 and CD7, the absence or weak expression of CD5, negativity of CD1a and/or CD8 and the expression of a myeloid lineage or stem cell antigen (CD13, CD33, CD11b, CD65, CD34, HLA-DR, CD117). T-ALL Egil III is characterized by CD3, CD7, CD1a and CD4 positivity

	ETP-ALL	T-ALL
Debut medullary blasts	51%	84%
Classification	EGIL T-I/II	EGIL T III
Immunophenotype	cyCD3 + + + CD34 + CD7 + CD11a + CD2 + CD117 + + CD1a- CD13- CD5- CD38 + + + CD8 + CD56 + + CD33 + HLA- DR +	cyCD3 + + + CD34- CD7 + + + CD11a + + + CD2 + CD117- CD1a + CD4 + + CD5 + + + CD38 + + + CD8- CD56- CD33- HLA- DR-
Allogeneic HSCT	YES	NO
Relapse	YES	NO
Immunophenotype after relapse	cyCD3 + + + CD34 + CD7 + + CD11a + CD2 + + CD117- CD1a- CD13 + CD5 + CD38 + + + CD8- CD56 ± CD33 + HLA- DR-	
Genetic hallmarks	Complete numerical aneuoploidies Chromosome 4 long arm (q) deletion −16;18 (der) unbalanced translocation	PICALM::MLLT10 fusion (not clinically significant)
Therapy	AIEOP-BFM 2017	AIEOP-BFM 2017

Low positive = + Medium positive = + + High positive = + + + according to the central testing laboratory validated by the AIEOP BFM 2017 protocol

## Conclusion

This case underscores the aggressive, chemoresistant nature of ETP-ALL and its distinct molecular profile[2]. Cytogenetic progression toward complex karyotype with 4q deletions and t(16;18) supports the concept of clonal evolution under therapeutic pressure (Fig. 1) [36]. Molecular analysis confirmed a BCL2-driven survival advantage, further underscored by reduced MFN2 and increased MARCH5, with concurrent C-MYC upregulation and BIM suppression. The Th17-like phenotype and decreased STING suggest an immunosuppressive microenvironment favoring leukemic persistence (Fig. 2).

Our findings corroborate emerging evidence of BCL2 dependence in ETP-ALL, supporting venetoclax as a rational, targeted therapy in relapsed/refractory cases (Fig. 3) [14]. Moreover, this study identifies candidate molecular markers IL23R, SGK1, RANBP1, MARCH5 with potential prognostic and therapeutic significance[29, 37].

Further studies are warranted to dissect the regulatory networks sustaining BCL2 overexpression, clarify post-transcriptional mechanisms stabilizing survival proteins, and explore strategies to modulate the immune microenvironment, ultimately improving outcomes in this high-risk leukemia subtype

## Supplementary Information

Below is the link to the electronic supplementary material.

Supplementary file1 (DOCX 533 kb)

## Data Availability

No datasets were generated or analysed during the current study.

## Abbreviations:

β-ACTIN: Actin beta
6MP: 6-Mercaptopurine
BCL2: BCL2 Apoptosis Regulator
BIM: BCL2 Like 11
BRAF: B-Raf Proto-Oncogene, Serine/Threonine Kinase
C-MYC: MYC Proto-Oncogene, BHLH Transcription Factor
CD45: Protein Tyrosine Phosphatase Receptor Type C
CPM: Cyclophosphamide
DEXA: Dexamethasone
DNMT3A: DNA Methyltransferase 3 Alpha
DNR: Daunorubicin
EP300: E1A Binding Protein P300
ETV6: ETS Variant Transcription Factor 6
EZH2: Enhancer Of Zeste 2 Polycomb Repressive Complex 2 Subunit
FLT3: Fms Related Receptor Tyrosine Kinase 3
FOXP3: Forkhead Box P3
G-CSF: Granulocyte colony stimulating factor
GAPDH: Glyceraldehyde-3-Phosphate Dehydrogenase
GATA3: GATA Binding Protein 3
HD-ARA-C: High-dose cytarabine
HD-MTX: High-dose methotrexate
HPRT1: Hypoxanthine Phosphoribosyltransferase 1
HR: High-risk block consolidation
HSCT: Hematopoietic Stem Cell Transplantation
IFO: Ifosfamide
IL17A: Interleukin 17A
IL23R: Interleukin 23 Receptor
IL7R: Interleukin 7 Receptor
JAK2: Janus Kinase 2
KMT2A: Lysine Methyltransferase 2A
KRAS: KRAS Proto-Oncogene, GTPase
LMO2: LIM Domain Only 2
LYL1: LYL1 Basic Helix-Loop-Helix Family Member
MARCH5: Membrane Associated Ring-CH-Type Finger 5
MEF2C: Myocyte Enhancer Factor 2C
MFN2: Mitofusin 2
MODS: Multiple Organ Dysfunction Syndrome
MRD: Minimal/measurable residual disease
NRAS: NRAS Proto-Oncogene, GTPase
PEG L-ASP: PEG-L-asparaginase
PRES: Posterior Reversible Encephalopathy Syndrome
RANBP1: RAN Binding Protein 1
RORγt: Retinoid-Related Orphan Receptor Gamma t
RUNX1: RUNX Family Transcription Factor 1
SGK1: Serum/Glucocorticoid Regulated Kinase 1
STING1: Stimulator Of Interferon Response cGAMP Interactor 1
TBI: Total body irradiation
VCR: Vincristine
VDS: Vindesine
VP-16: Etoposide
