# Supplementary material for: Cytogenetic and molecular characterization of an atypical ETP-ALL case with BCL2 dependency: therapeutic implications for Venetoclax use
Source: Mol Biol Rep. 2025 Sep 18;52(1):922. doi: 10.1007/s11033-025-10979-1 (PMC12446117; doi:10.1007/s11033-025-10979-1)
Supplement: Supplementary file 1 — Supplementary file1 (DOCX 533 kb) [file 11033_2025_10979_MOESM1_ESM.docx]

***SUPPLEMENTARY FILES***

**Supplementary Table 1.** Clinical, immunophenotypic, and laboratory characteristics at different disease stages.

LEGEND

LOW POSITIVE = +

MEDIUM POSITIVE = ++

HIGH POSITIVE = +++

according to the central testing laboratory validated by the AIEOP BFM 2017 protocol.

**Supplementary Table 2: reagents and resources.**

| REAGENT or RESOURCE | SOURCE | IDENTIFIER |
| --- | --- | --- |
| **Antibodies** | | |
| RANBP1 | Santa Cruz Biotechonology | Sc-374352 |
| SGK1 | Millipore Corporation, CA, USA | #07-315 EDM |
| IL-23R | Thermo Fischer Scientific | #PA5-113004 |
| BCL-2 (N-19) | Santa Cruz Biotechonology | SC-492 |
| C-MYC (N-262) | Santa Cruz Biotechonology | SC-764 |
| STING (D2P2F) | Cell Signaling Technology | #13647 |
| Mitofusin-2 (D1E9) | Cell Signaling Technology | 11925T |
| MARCH5 | Life Technologies - Thermo Fisher Scientific | PA5-25584 |
| BIM(C34C5) | Cell Signaling Technology | #2933 |
| β-actin | SIGMA | A5441 |
| Anti-mouse IgG HRP-linked | Cell Signaling Technology | #7076 |
| Anti-rabbit IgG HRP-linked | Cell Signaling Technology | #7074 |
| **Oligonucleotides** | | |
| hsa-IL-23R  fwd: 5’- TACTGGCAGCCTTGGAGTTCA -3’  rev: 5’- TAAGGTGCCCTGTAGAGATGGA- 3’ |  | N/A |
| hsa-IL-17A  fwd:5’-TACAACCGATCCACCTCACCTT-3’  rev: 5’- ACTTTGCCTCCCAGATCACAGA- 3’ | Life Technologies - Thermo Fisher Scientific | (Spagnuolo et al.) |
| hsa-RORC  fwd: 5’- GCAGCGCTCCAACATCTTCT-3’  rev:5’- ACGTACTGAATGGCCTCGGT-3’ | Life Technologies - Thermo Fisher Scientific | N/A |
| hsa-SGK1  fwd: 5’- GGCACCCTCACTTACTCCAG- 3’  rev: 5’- GGCAATCTTCTGAATAAAGTCGTT- 3’ | Life Technologies - Thermo Fisher Scientific | (Dattilo et al.) |
| hsa-RANBP1  fwd: 5’- ATGCGGGCAAAACTGTTCCGAT- 3’  rev: 5’- ATGGCCCCTTTCTCCTTGTGCT- 3’ | Life Technologies - Thermo Fisher Scientific | (Dattilo et al.) |
| hsa-BCL2  fwd: 5’- ATC GCC CTG TGG ATG - 3’  rev: 5’- CCA GGA GAA ATC AAA - 3’ | Eurofins Genomics | N/A |
| hsa-STING  fwd: 5’- GGGCTGGCATGGTCATATTACA - 3’  rev: 5’- GAATATACAGCCGCTGGCTCAC - 3’ | Life Technologies - Thermo Fisher Scientific | N/A |
| hsa-CD45  fwd: 5’- GGAGACTATCCTGGAGAACCCT - 3’  rev: 5’- CAACAAGCTCCTGCTGTTCATC - 3’ | Life Technologies - Thermo Fisher Scientific | N/A |
| hsa-FOXP3  fwd: 5’- GACAGGCCACATTTCATGCAC- 3’  rev: 5’- TGGTGTGAGGCTGATCATGG- 3’ | Eurofins Genomics | N/A |
| hsa-HPRT1  fwd: 5’- TGACACTGGCAAAACAATGCA- 3’  rev: 5’- GGTCCTTTTCACCAGCAAGCT- 3’ | Life Technologies - Thermo Fisher Scientific | (Vandesompele et al.) |
| MARCH5 | Life Technologies - Thermo Fisher Scientific | Hs01546967 |
| MITOFUSIN-2 | Life Technologies - Thermo Fisher Scientific | Hs00208382 |
| GAPDH | Life Technologies - Thermo Fisher Scientific | Hs03929097_g1 |

**
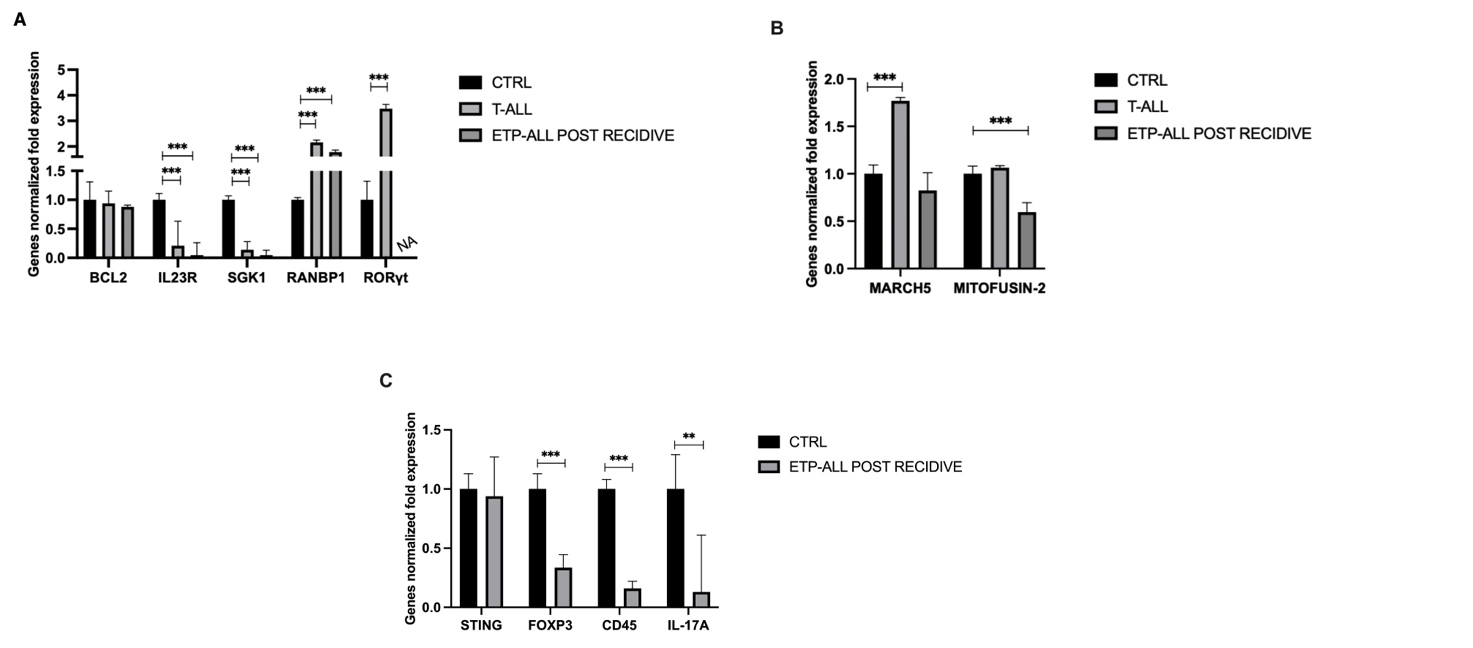
**

**Supplementary Figure 1. Transcripts analyses during ETP-ALL pathological stages A-B**: qPCR analysis to assess the expression of BCL2, IL23R, SGK1, RANBP1 and RORγt **(A)**; MARCH5 and MITOFUSIN-2 **(B)** in primary bone morrow lymphocyte, derived from a Healthy control, classical T-ALL and ETP-ALL samples. Equal loading was verified by means of GAPDH (n=5 qPCR). **C**: qPCR analysis to assess the expression of STING, FOXP3, CD45, IL17 in primary bone morrow lymphocyte, derived from a Healthy control and ETP-ALL samples. Equal loading was verified by means of HPRT-1 (n=5 qPCR).


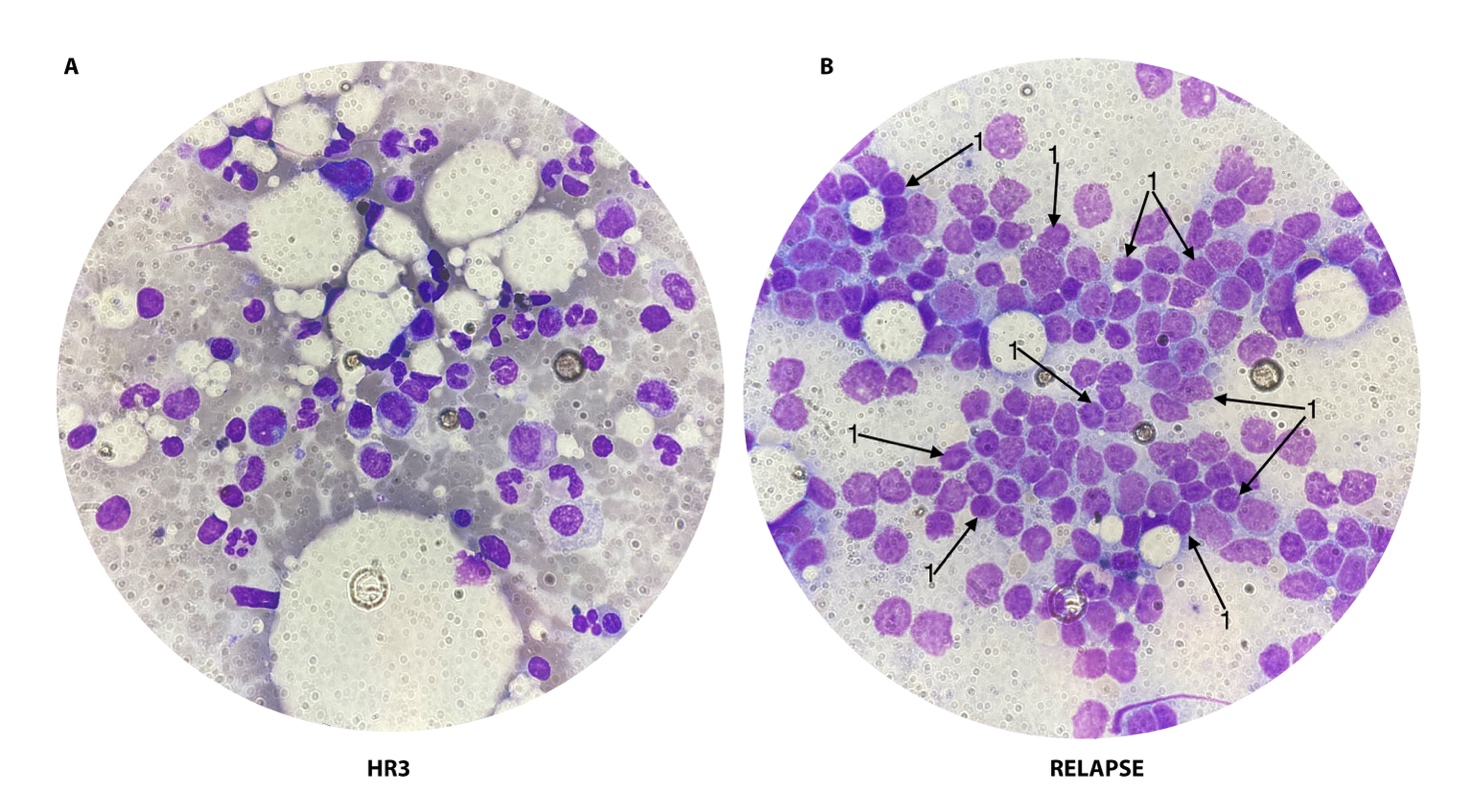
**Supplementary Figure 2. Bone marrow smears.** Bone marrow smears illustrating marrow cytology during the HR3 phase (**A**) and at relapse (**B**), stained with May-Grünwald-Giemsa using the Sysmex SP-10 automated instrument (Dasit). Label 1 indicates leukemic blasts;
